# Effects of Summer Heat on Adipose Tissue Activity in Periparturient Simmental Cows

**DOI:** 10.3390/metabo14040207

**Published:** 2024-04-06

**Authors:** Romana Turk, Nikola Rošić, Blanka Beer Ljubić, Silvijo Vince

**Affiliations:** 1 Department of Pathophysiology, Faculty of Veterinary Medicine, University of Zagreb, 10000 Zagreb, Croatia; 2 Veterinary Practice Jastrebarsko, 10450 Jastrebarsko, Croatia; 3 Laboratory of Internal Clinic, Faculty of Veterinary Medicine, University of Zagreb, 10000 Zagreb, Croatia; 4 Department for Reproduction with Clinic for Obstetrics, Faculty of Veterinary Medicine, University of Zagreb, 10000 Zagreb, Croatia; svince@vef.unizg.hr

**Keywords:** heat stress, lipid mobilization, transition period, non-esterified fatty acids, beta-hydroxy butyrate, leptin, adiponectin

## Abstract

Hot climate is one of the major factors affecting the dairy industry. Heat stress could be responsible for decreased feed intake and consequently leads to alterations in energy metabolism, particularly during late pregnancy and early lactation. This study aimed to assess the effects of summer heat on adipose tissue activities during the periparturient period in Simmental cows. Two groups of cows were involved: heat-stressed cows (n = 12) that calved from June to August and thermoneutral cows (n = 12) that calved from October to December. Blood samples were taken from each cow during the periparturient period: 21 and 7 days before calving and 8, 16, 24, 32, and 40 days after calving. Glucose, beta-hydroxy butyrate (BHB), non-esterified fatty acids (NEFA), leptin (LP), and adiponectin (ADP) were measured in serum samples by commercial kits. Thermoneutral cows expressed higher degrees of lipomobilization syndrome than heat-stressed cows, indicated by significantly higher serum NEFA and BHB concentrations in the early lactation. Leptin levels were significantly decreased, while adiponectin was increased in heat-stressed cows compared to thermoneutral ones. The results indicated that heat-stressed cows during the periparturient period mobilized less fat from adipose tissue to reduce the heat generation by fatty acid oxidation.

## 1. Introduction

Global warming, driven by climate change, has led to rising temperatures and more frequent and severe heatwaves. Dairy cows are particularly vulnerable to heat stress. High thermal environments can cause a decreased feed intake in cows, leading to decreased milk production [1,2]. This could be mediated by metabolic stress, which compromises the energy metabolism of cows, especially during the periparturient period [2,3]. Prolonged exposure to heat stress can weaken the immune system of dairy cows [4,5]. A compromised immune system makes cows more susceptible to diseases and infections, further affecting their productivity and posing long-term impacts on the dairy industry [2]. 

During the transition from late pregnancy to early lactation, dairy cows experience a significant increase in energy requirements. This increase is primarily driven by the need for both fetal growth and milk synthesis that dramatically exceed the amount of energy the cow can obtain from dietary sources. The substantial surge in energy requirements during this period often leads to a Negative Energy Balance (NEB). The metabolic adaptation to NEB requires interactions of complex metabolic pathways affecting mostly glucose and lipid metabolism occurring in the liver, adipose tissue, and other tissues. Heat stress can even exacerbate the detrimental effects of NEB, potentially leading to more severe disorders and systemic inflammation. Intense lipid mobilization from adipose tissue to meet energy requirements leads to a substantial release of non-esterified fatty acids (NEFA) into circulation, enabling the redirection of glucose for both fetal metabolism and lactose synthesis. Increased lipid mobilization and NEFA oxidation in the liver could be associated with oxidative stress and inflammatory response [6,7]. 

During heat stress, cows tend to generate less metabolic heat, leading to a decrease in lipid mobilization and fatty acid oxidation in the liver [8,9]. Consequently, NEFA and BHB concentrations are expected to be lower in early lactation in heat-stressed cows compared to thermoneutral ones [9,10].

Adipose tissue is the major source of energy supply in transition dairy cows and plays a key role in the integration of energy metabolism. Besides serving as an energy store, adipose tissue is also considered an important endocrine organ playing a significant role in systemic homeostasis, particularly in glucose and lipid metabolism, insulin resistance, and immunity and inflammation [11]. These roles are achieved by the synthesis and secretion of numerous bioactive proteins collectively called adipokines [12]. Among them, adiponectin (ADP) and leptin (LP) are the most abundant hormones playing a role in metabolic stress in dairy cows during the transition period [13,14,15,16]. Previous studies have shown that adiponectin and leptin levels in the early postpartum period of cows are inversely related to NEFA and BHB concentrations [13,14,15,16,17]. The dynamics of leptin and adiponectin levels in heat-stressed cows during the transition period are still poorly understood. The aim of this study was therefore to investigate the effects of heat stress on adipose tissue activity: lipid mobilization and adipokine dynamics (leptin and adiponectin) during the transition period in cows.

## 2. Materials and Methods

### 2.1. Animals and Study Design

This study included twenty-four healthy Simmental cows aged 2 to 5 years. The animals were located on a dairy farm situated in Zagreb County. According to the season, the cows were divided into two groups. The first group (n = 12) consisted of cows with calving dates in the summer period (June, July, August), while the second group (n = 12) consisted of cows with calving dates in the autumn period (October, November, December). Cows in the summer group had a body condition score (BCS) ranging from 3.0 to 3.5 with average milk production of 5760 kg during their previous lactation. Cows included in the autumn group had a BCS of 3.2–3.7 with average milk production of 5920 kg during their previous lactation. Both groups of cows were in optimal body condition. There were no differences in milk production between groups. Cows were housed in a partially open free-stall barn designed with roof ventilation to facilitate the circulation of fresh air and the removal of excess moisture and heat. The cows were kept freely with sufficient space for movement and elevated individual resting beds for rest. All cows were provided with high-quality hay and silage ad libitum, with the addition of concentrate, the quantity of which was individually dosed for each animal using a ‘computerized feeding’ system. Additionally, 20 days before the expected calving, the cows received 2 kg of concentrate daily, and after calving, an additional 1 kg per day was added, reaching a total of 8 kg of concentrate per day. From the 15th day, the daily quantity of concentrate was increased to 10 kg. The concentrate consisted of 70% ground maize grain and 30% supplementary cattle feed mixture for dairy cows with 32% protein. The analytical composition of the concentrate is reported in Table 1.

During the study, air temperature, relative humidity, and air movement within the barn were recorded during each blood-sampling event. These data were collected using a climate-measuring device (Testo 445, Melrose, MA, USA). Subsequently, a temperature–humidity index (THI) was computed from the gathered data, following the equation established by the National Research Council (U.S.). Vital parameters, i.e., rectal temperature and heart and respiratory rates, were recorded as well at each blood sampling. Rectal temperature was measured by a digital thermometer while heart and respiratory rates were measured using a stethoscope.

The study was approved by the Ethical Committee of the Faculty of Veterinary Medicine University of Zagreb (Reference number 251-61-01/139-15-2).

### 2.2. Blood Sampling and Laboratory Assays

Blood samples were obtained from *v. coccygea* and collected into Vacutainer tubes without an anticoagulant containing a clot activator on seven occasions: 21 and 7 days before calving and 8, 16, 24, 32, and 40 days after calving. Following a two-hour clotting period at room temperature, samples were centrifuged at 3000× *g* for 30 min. Serum samples were stored at −80 °C until analysis. 

The concentrations of glucose, beta-hydroxy butyrate (BHB), and non-esterified fatty acids (NEFA) were analyzed using the commercial kits (Beckman Coulter Biomedical Ltd., Ireland, and Randox, Ireland, respectively) on the Beckman Coulter AU 640 biochemical analyzer (Beckman Coulter Biomedical Ltd., Ireland). Leptin and adiponectin concentrations were assayed by ELISA kits specific for bovines ((MyBioSource, Inc., San Diego, CA, USA). The bovine leptin antibody ELISA kit applies the competitive enzyme immunoassay technique utilizing a leptin antigen and a leptin-Ab-HRP conjugate. The bovine total adiponectin (ADPN) ELISA kit is a quantitative sandwich ELISA assay using purified bovine ADPN antibody to coat microelisa stripplate wells to make a solid-phase antibody and an ADPN antibody that were labeled with HRP. The color change was measured spectrophotometrically at a wavelength of 450 nm.

### 2.3. Statistical Analysis

Statistical analysis of the data was performed using SAS 9.4 software (SAS Institute Inc., Cary, NC, USA). Normal data distribution was tested using the PROC TRANSREG module. When the presumptions of the normal distribution of the analyzed dependent variables were disturbed and in the case of heteroscedasticity of variances, the transformation of variables was performed by log or exponential transformation. A quick testing of dependent variables was performed by multivariance analysis of variance based on the criterion of Wilks’ lambda using the GLM procedure. The main model was performed by the mixed module (PROC MIXED) and it included the fixed effect of the group, period, and their interaction. The random effect of an animal with repeating measurements (animal identification number) over time was included in the model. The decisions of which type of variance–covariance structure was used in the model were based on the SAS criteria for evaluating model fitting (AIC and BIC). The multiple comparison test of the least-square means with Tukey’s correction was performed using the SLICE option to compare each group level within the period. The level of statistical significance was set at *p* < 0.05. The results were expressed as least squares mean (LSM) and standard error of the mean. After analysis, if the transformation was performed, the data were reversed to their original values.

## 3. Results

### 3.1. Temperature–Humidity Index and Airflow

The temperature–humidity index (THI) was significantly higher (*p* < 0.0001) in the summer group at each sampling time compared to the autumn group (Table 1), indicating mild to moderate heat stress in cows in the summer season [18]. In addition to the higher air temperature, the airflow was significantly lower (*p* < 0.0001) in summer than in autumn, also contributing to the heat stress (Table 2). 

### 3.2. Animal Vital Parameters (Rectal Temperature, Heart and Respiratory Rates)

Rectal temperature was significantly higher (*p* < 0.05) in the summer group at days −21, −7, 8, 16, 24, and 32 relative to calving compared to the cows in autumn, while there was no significant difference at day 40 after calving between groups (Table 3). Respiratory rate was significantly higher (*p* < 0.001) in cows during summer at days −7, 8, 16, 24, 32, and 40 relative to calving than in cows during autumn on the same days. Heart rate was only higher in the summer group on the 16th day after calving compared to the autumn group (*p* < 0.05), Table 3. These results indicated that a hot environment with increased ambient temperature and decreased air movement changed the vital parameters of cows during the summer months.

### 3.3. Serum Glucose, Nonesterified Fatty Acid (NEFA) and Betahydrohy-Butyrate (BHB) Concentrations

In the autumn group, the glucose levels showed a significant decrease postpartum (*p* < 0.01) on the 8th, 16th, 24th, and 32nd days postpartum compared to the value recorded 21 days before calving (Table 4). This decrease indicates a reduction in blood glucose availability in cows after calving. Within the summer group, no significant differences in serum glucose levels were observed during the periparturient period (*p* > 0.05), indicating a consistent availability of blood glucose in early lactation. Furthermore, there were no statistically significant differences (*p* > 0.05) in glucose concentrations between the summer and autumn groups at any of the sampling points (Table 4).

In the autumn period, NEFA concentrations exhibited a significant increase (*p* < 0.001) after calving on the 8th, 16th, 24th, and 32nd days, in contrast to the values recorded at 21 days prior to calving, as well as at 40 days postpartum (Table 4). This observation indicates substantial lipid mobilization from adipose tissue during the early stages of lactation. During the summer period, NEFA concentration displayed only a significant increase closely around calving, from one week before to one week after calving, on days −7 (*p* < 0.05) and 8 (*p* < 0.001) relative to calving (Table 4), showing a different pattern of lipomobilization syndrome compared to that observed in cows during the autumn period. Comparing the equivalent sampling points between the summer and autumn groups, the NEFA levels were significantly lower in the autumn group on day −7 before calving but demonstrated a higher postpartum trend from the 16th to the 40th days after calving (Table 4).

During the autumn period, BHB concentrations exhibited a significant increase (*p* < 0.001) post-calving from the 8th to the 40th days when compared with the values on days −21 and −7 before calving. This aligns with the lipomobilization syndrome, as evidenced by the rise in NEFA concentrations postpartum in the autumn group. It also raises the possibility of ketosis since BHB levels reached the threshold for ketosis of 1.2 mmol/L on the 16th and 24th days of lactation (Table 4). On the contrary, in the summer group, there were no statistically significant changes in BHB levels during the periparturient period, which reflected a low degree of lipomobilization syndrome during summer. When comparing both groups, it is notable that the BHB levels were significantly higher in the autumn group on the 8th (*p* < 0.05), 16th (*p* < 0.001), and 24th (*p* < 0.01) days postpartum, in contrast to the summer group. This suggests that cows during the summer period mobilized less fat than their counterparts in the autumn (Table 4).

### 3.4. Serum Adiponectin (ADP) and Leptin (LP) Concentrations

Serum concentrations of ADP and LP exhibited distinct variations in response to different seasons.

**Figure 1:**
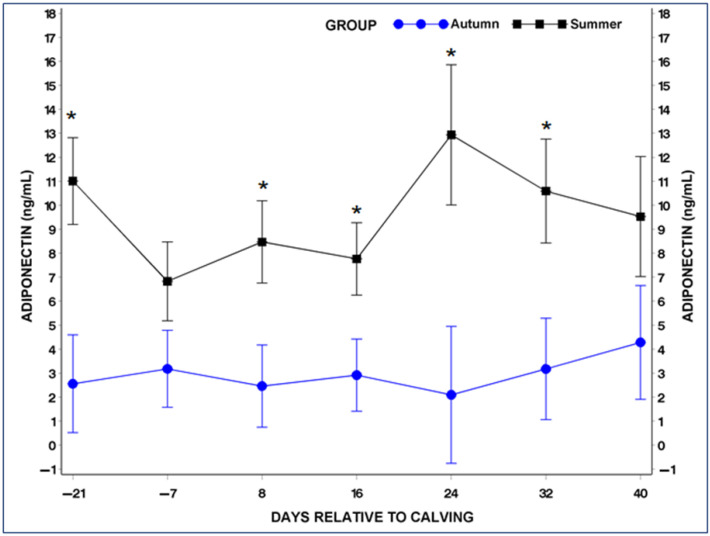
Serum adiponectin (ADP) concentrations in the summer and autumn groups during the periparturient period. * Statistical significant difference between the summer and autumn groups at the equivalent samplimg points (*p* < 0.05).

In general, the average serum ADP in cows during summer (9.4 ng/mL) was higher than in cows during autumn (2.9 ng/mL), although the difference was on the threshold of statistical significance (*p* = 0.053). Particularly, ADP levels were significantly higher (*p* < 0.05) in the summer group on day −21 before calving and on the 8th, 16th, 24th, and 32nd days postpartum, in contrast to the same days in the autumn group (Figure 1).

**Figure 2:**
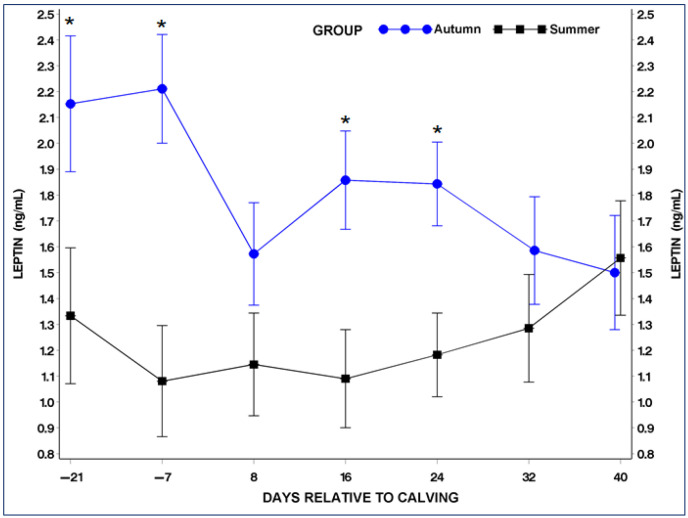
Serum leptin (LP) concentrations in the summer and autumn groups during the periparturient period. * Statistical significant difference between the summer and autumn groups at the equivalent samplimg points (*p* < 0.05).

Overall, the average serum LP in cows during summer (1.2 ng/mL) was significantly lower (*p* < 0.05) than in cows during autumn (1.8 ng/mL), as depicted in Figure 2. Particularly, the LP level was significantly higher (*p* < 0.05) in the autumn group on days −21 and −7 before calving and on the 16th and 24th days postpartum, in contrast to the same days in the summer group (Figure 2). In the autumn group, LP concentrations were higher before calving than postpartum, although these differences were not statistically significant (*p* > 0.05), as illustrated in Figure 2. No significant variations in LP levels were observed in the summer group during the periparturient period (Figure 2).

### 3.5. Correlation between THI, NEFA, BHB, ADP, and LP

The correlations between THI, NEFA, BHB, ADP, and LP are presented in Table 5. The concentrations of BHB and leptin were significantly negatively correlated with THI, indicating that during heat stress, there was less BHB and leptin production and thus a lower degree of lipomobilization syndrome. The reduction in leptin ensured the preservation of glucose and energy in cows with pronounced energy deficit On the contrary, adiponectin concentration significantly positively correlated with THI, demonstrating increased adiponectin expression during heat stress, consequently increased insulin sensitivity, and decreased lipid mobilization in adipose tissue. Concentrations of NEFA and BHB showed a significant positive correlation that demonstrated increased ketogenesis due to increased NEFA mobilization from adipose tissue and vice versa. BHB concentration showed a significant positive correlation with leptin and a negative one with adiponectin, pointing out that BHB is related to adipose tissue activity. Leptin and adiponectin showed a significant negative correlation, indicating the opposite expression in cows during the periparturient period.

## 4. Discussion

This study has shown that adipose tissue metabolism in periparturient cows is affected by heat stress compared to non-heat-stressed cows. During the hot season, cows generate a significant amount of metabolic heat and absorb additional heat from their environment, especially during lactation. High ambient temperatures and increased relative humidity prevent the cows from dissipating the accumulated heat, making them susceptible to heat stress. This results in elevated body temperature, leading to reduced feed intake and consequently decreased milk production, which has been well documented previously [8,19,20] In our study, cows showed increased rectal body temperature and respiratory rate in summer, indicating heat stress, which is consistent with other studies [21,22]. The temperature–humidity index (THI) is a widely recognized indicator for evaluating heat stress levels, whereby a THI surpassing 72 suggests the adverse effects of a hot environment on body homeostasis and production [18,23,24]. In our study, THI in the summer period was between 77 and 82 compared to 57–62 in the autumn, indicating that cows during the summer period were exposed to mild to moderate heat stress according to criteria widely proposed [18,23,24]. 

There were no significant differences in glucose concentration between the summer and autumn groups in this study. However, in the autumn group, there was a drop in glucose after partus and in the early postpartum period, while in the summer group, the glucose levels were stable during the periparturient period. In addition, cows during the thermoneutral period expressed a higher degree of lipomobilization syndrome after calving, indicated by higher levels of NEFA and BHB in the early lactation, compared to heat-stressed cows, which showed a lower degree of lipid mobilization. In our previous study [25] conducted on heifers, the response to heat stress was characterized by higher NEFA levels during the transition period. The reason why heifers responded differently to heat stress compared to cows could be attributed to greater body surface area relative to body mass, as proposed by West et al. [8]. This characteristic may make them less susceptible to heat stress.

Similar results to the current study were obtained by Amaral et al. [10] and Lamp et al. [9], whose studies found significantly higher NEFA levels in cooled cows after calving compared to heat-stressed cows. These results could be associated with the assumption that during heat stress, the mobilization of adipose tissue is suppressed, resulting in lower production of metabolic heat generated by fatty acid oxidation [2,3,8,26,27]. Thus, heat-stressed dairy cows reduce metabolic heat production in late pregnancy and early lactation by reduced lipid mobilization and consequently decreased BHB production [9]. This is indicated in our study by the reversed significant correlation between THI and BHB levels, as shown in Table 5. In our study, heat-stressed cows demonstrated increased NEFA levels only before calving, while BHB was unchanged during the periparturient period. Similar results were also suggested by Lamp et al. [9]. This is a compensatory mechanism of cows to produce less heat by fatty acid oxidation in a high thermal environment. However, the exact mechanisms by which heat-stressed cows might reduce heat production by decreased lipolysis and fatty acid oxidation remain to be elucidated. 

Adipose tissue not only serves as a lipid store to supply the body with additional energy but is also a highly active metabolic and endocrine organ secreting hormone-like mediators generally known as adipokines, including leptin, adiponectin, and cytokines such as tumor necrosis factor α (TNFα) and interleukin 6 (IL-6), among others [11,28]. Among many physiological functions, adipokines regulate glucose and lipid metabolism, insulin sensitivity, reproductive function, appetite and body weight control, angiogenesis and blood pressure, and immunity and inflammation [11]. Leptin belongs to the IL-6 family of cytokines, which induces lipolysis and fatty acid oxidation during reduced feed intake and stimulates energy utilization [15]. In addition, it decreases appetite and consequently reduces feed intake. It is partly regulated by insulin; it decreases when insulin levels are low and increases with feeding, i.e., in response to increased insulin levels [11]. Other hormones are also implicated in leptin regulation, such as estrogens and glucocorticoids, which increase leptin expression and secretion, while growth hormone (GH) and androgens decrease leptin concentration [28]. In our study, leptin concentration was significantly lower in heat-stressed cows compared to thermoneutral ones. This could be a consequence of decreased insulin levels due to decreased feed intake in heat-stress conditions. There are few studies that have investigated the effects of heat stress on leptin production in cows. Min et al. [21] demonstrated no differences in leptin concentrations during mid-lactation between heat-stressed and thermoneutral cows. The reason for the results differing from ours is probably due to the different physiological periods studied in the cows, as the mid-lactation period is not characterized by a high degree of adaptive metabolism, as is the case in the periparturient period. This could implicate that processes of metabolic adaptation to high-energy demands might be detrimental factors influencing leptin production and concentration. Opposite results to ours were obtained by Bernabucci et al. [23] and Stefanska et al. [29], who observed increased leptin concentrations and NEFA and BHB in heat-stressed cows in the early lactation. Ehrhardt et al. [30] demonstrated a significant reduction in plasma glucose after leptin administration in early lactating dairy cows in normal thermal conditions. We obtained similar results in thermoneutral cows, where glucose and leptin concentrations were lower after parturition compared to values observed in late pregnancy. 

Adiponectin has been widely suggested to have a role in energy homeostasis regulating lipid and carbohydrate metabolism. It has been found in humans and mice that it can improve insulin sensitivity and thus stimulate glucose uptake and utilization by skeletal muscle and adipose tissue. Caloric deficit leads to increased adiponectin levels, which in turn increase insulin sensitivity. Additionally, adiponectin could stimulate fatty acid oxidation and express anti-inflammatory and antioxidant effects [28,31,32]. In our study, adiponectin levels were significantly higher in heat-stressed cows than in thermoneutral cows. This is also demonstrated by the significant positive correlation between THI and adiponectin. These findings could indicate a caloric deficit in heat-stressed cows due to reduced feed intake and consequently increased glucose utilization mediated by increased insulin sensitivity. Adiponectin concentration significantly inversely correlated with NEFA and BHB levels in our study. Thermoneutral cows had lower adiponectin concentrations and higher NEFA and BHB levels than heat-stressed cows. This illustrated that thermoneutral cows had lower insulin sensitivity due to lower adiponectin levels and thus shifted the energy metabolism to increased fatty acid oxidation. Cows with higher NEFA levels are predisposed to inflammatory diseases. Our results are in accordance with Mann et al. [13], who demonstrated that hyperketonemic cows during the periparturient period had lower adiponectin concentrations compared to those with non-hyperketonemic episodes. Kabara et al. [14] also demonstrated the inverse correlation between adiponectin and NEFA during the periparturient period. They also evidenced that the bovine monocyte exposure to adiponectin reduced their inflammatory response. This suggests that reduced plasma adiponectin during the periparturient period could predispose dairy cows to increased inflammatory responses. Lower plasma adiponectin associated with increased lipolysis and the decreased insulin sensitivity of peripheral tissues around parturition in dairy cows were also suggested by Singh et al. [16]. The precise pathways that mediate the metabolic effects of adiponectin in periparturient cows, particularly in heat-stress conditions, remain poorly understood. Further work is needed to elucidate the exact mechanisms by which leptin and adiponectin mediate adaptive metabolism in heat-stressed cows during the periparturient period.

## 5. Conclusions

This study demonstrated different adaptive mechanisms and adipose tissue activities between thermoneutral and heat-stressed cows during the periparturient period. In heat-stressed cows, the rate of adipose tissue mobilization was limited, with lower circulating NEFA concentrations and consequently lower concentrations of BHB in early lactation compared to thermoneutral cows. On the contrary, thermoneutral cows maintained their metabolic homeostasis during the periparturient period by adaptive metabolism involving the mobilization of endogenous energy reserves from adipose tissue leading to increased NEFA and BHB concentration after parturition. The hormonal activity of adipose tissue also changed between heat-stressed and thermoneutral cows. Heat-stressed cows responded with significantly lower leptin concentrations and higher adiponectin levels during the periparturient period compared to thermoneutral cows. Therefore, different patterns in the dynamics of adipose tissue activities could help cows adapt to summer heat by reducing lipid mobilization to decrease metabolic heat production and by decreasing leptin and increasing adiponectin levels to improve insulin sensitivity and thus stimulate glucose uptake and utilization by peripheral tissues. The exact pathways mediating the involvement of leptin and adiponectin in the processes of the metabolic adaptation of cows in heat-stress conditions still need to be better investigated.

## Figures and Tables

**Table 1:** Analytical composition of the concentrate.

Ingredients	%
Crude protein	32
Crude fats	2
Crude fibers	9
Calcium	2.5
Phosphorus	1.2
Potassium	1
Magnesium	0.25

**Table 2:** Temperature–humidity index (THI) and airflow (m/s) in the summer and autumn groups during the periparturient period.

Days Relative to Calving	THI		Airflow (m/s)	
	Autumn	Summer	Autumn	Summer
−21	60	78 *	0.45	0.18 *
−7	59	79 *	0.45	0.17 *
8	58	80 *	0.45	0.18 *
16	62	82 *	0.45	0.18 *
24	60	81 *	0.48	0.18 *
32	57	77 *	0.48	0.19 *
40	57	77 *	0.48	0.20 *

* Statistically significant differences between the summer and autumn groups (*p* < 0.0001).

**Table 3:** Rectal temperature (°C) and heart (beats/minute) and respiratory rates (breaths/minute) in the summer and autumn groups during the periparturient period.

Days Relative to Calving	Rectal Temp. (°C)		Heart Rate (Beats/Minute)		Respiratory Rate (Breaths/Minute)	
	Autumn	Summer	Autumn	Summer	Autumn	Summer
−21	38.7	39.1 *	90.4	88.6	31.4	37.1
−7	38.8	39.2 *	85.9	93.8	29.6	47.4 **
8	38.7	39.2 *	83.0	86.7	25.8	54.3 ***
16	38.8	39.5 *	84.3	91.8 *	27.3	63.9 ***
24	38.7	39.3 *	86.0	91.8	27.3	66.2 ***
32	38.6	39.0 *	83.9	84.2	26.1	52.0 ***
40	38.7	38.8	85.3	87.0	26.3	42.6 ***

Statistically significant differences between the summer and autumn groups: * *p* < 0.05; ** *p* < 0.001; *** *p* < 0.0001.

**Table 4:** Serum glucose, nonesterified fatty acid (NEFA), and betahydroxy-butyrate (BHB) concentrations in the summer and autumn groups during the periparturient period.

Days Relative to Calving	Glucose (mmol/L)		NEFA (mmol/L)		BHB (mmol/L)	
	Autumn	Summer	Autumn	Summer	Autumn	Summer
−21	3.3 ^a^	3.0	0.13 ^ac^	0.14 ^a^	0.54 ^a^	0.51
−7	3.2 ^ac^	2.9	0.11 ^bc^	0.39 ^b^*	0.57 ^a^	0.44
8	2.8 ^bc^	2.9	0.67 ^b^	0.51 ^b^	0.96 ^b^	0.58 *
16	2.8 ^b^	2.7	0.57 ^b^	0.31 ^a^*	1.21 ^b^	0.55 ***
24	2.7 ^b^	2.8	0.46 ^b^	0.28 ^a^*	1.34 ^b^	0.70 **
32	2.7 ^b^	2.8	0.47 ^b^	0.24 ^a^*	1.13 ^b^	0.63
40	2.9 ^a^	3.2	0.28 ^ac^	0.10 ^a*^	1.17 ^b^	0.53

^a,b,c^ Values with different superscripts are significantly different (*p* < 0.05/*p* < 0.001). Statistically significant differences between the summer and autumn groups: * *p* < 0.05; ** *p* < 0.001; *** *p* < 0.0001.

**Table 5:** Spearman coefficients of correlation and *p* values between THI, NEFA, BHB, LP, and ADP in all samples.

	NEFA	BHB	LP	ADP
**THI**	n.s.	−0.34 <0.0001	−0.42 <0.0001	0.37 <0.0001
**NEFA**		0.44 <0.0001	n.s.	−0.29 <0.001
**BHB**			0.31 <0.0001	−0.44 <0.0001
**LP**				−0.67 <0.0001

n.s.—non-significant.

## Data Availability

The row datasets are not available due to privacy. Further inquiries can be directed to the corresponding author.
